# Supplementary material for: Resolving the full spectrum of human genome variation using Linked-Reads
Source: Genome Res. 2019 Apr;29(4):635–45. doi: 10.1101/gr.234443.118 (PMC6442396; doi:10.1101/gr.234443.118)
Supplement: Supplemental Material [file supp_gr.234443.118_Supplemental_longranger-2.2.zip › longranger-2.2.tar.bz2!/lr2.2-public/lib/stan/cmdstan/stan/doc/api/html/rev_2operators_2operator__addition_8hpp_source.html]

Stan: src/stan/agrad/rev/operators/operator\_addition.hpp Source File


|  |  |
| --- | --- |
|  | Stan  2.5.0  probability, sampling & optimization |


- Main Page
- Related Pages
- Namespaces
- Classes
- Files

- File List
- File Members

All Classes Namespaces Files Functions Variables Typedefs Enumerations Enumerator Friends Macros Pages

- src
- stan
- agrad
- rev
- operators

operator\_addition.hpp

Go to the documentation of this file.

1 #ifndef STAN\_\_AGRAD\_\_REV\_\_OPERATORS\_\_OPERATOR\_ADDITION\_HPP

2 #define STAN\_\_AGRAD\_\_REV\_\_OPERATORS\_\_OPERATOR\_ADDITION\_HPP

3

4 #include <stan/agrad/rev/var.hpp>

5 #include <stan/agrad/rev/internal/vv\_vari.hpp>

6 #include <stan/agrad/rev/internal/vd\_vari.hpp>

7 #include <boost/math/special\_functions/fpclassify.hpp>

8

9 namespace stan {

10  namespace agrad {

11

12  namespace {

13  class add\_vv\_vari : public op\_vv\_vari {

14  public:

15  add\_vv\_vari(vari\* avi, vari\* bvi) :

16  op\_vv\_vari(avi->val\_ + bvi->val\_, avi, bvi) {

17  }

18  void chain() {

19  if (unlikely(boost::math::isnan(avi\_->val\_)

20  || boost::math::isnan(bvi\_->val\_))) {

21  avi\_->adj\_ = std::numeric\_limits<double>::quiet\_NaN();

22  bvi\_->adj\_ = std::numeric\_limits<double>::quiet\_NaN();

23  } else {

24  avi\_->adj\_ += adj\_;

25  bvi\_->adj\_ += adj\_;

26  }

27  }

28  };

29

30  class add\_vd\_vari : public op\_vd\_vari {

31  public:

32  add\_vd\_vari(vari\* avi, double b) :

33  op\_vd\_vari(avi->val\_ + b, avi, b) {

34  }

35  void chain() {

36  if (unlikely(boost::math::isnan(avi\_->val\_)

37  || boost::math::isnan(bd\_)))

38  avi\_->adj\_ = std::numeric\_limits<double>::quiet\_NaN();

39  else

40  avi\_->adj\_ += adj\_;

41  }

42  };

43  }

44

83  inline var operator+(const var& a, const var& b) {

84  return var(new add\_vv\_vari(a.vi\_,b.vi\_));

85  }

86

98  inline var operator+(const var& a, const double b) {

99  if (b == 0.0)

100  return a;

101  return var(new add\_vd\_vari(a.vi\_,b));

102  }

103

115  inline var operator+(const double a, const var& b) {

116  if (a == 0.0)

117  return b;

118  return var(new add\_vd\_vari(b.vi\_,a)); // by symmetry

119  }

120

121  }

122 }

123 #endif

boost::math::isnan

bool isnan(const stan::agrad::var &v)

Checks if the given number is NaN.

**Definition:** boost\_fpclassify.hpp:69

stan::agrad::operator+

fvar< T > operator+(const fvar< T > &x1, const fvar< T > &x2)

**Definition:** operator\_addition.hpp:14

vd\_vari.hpp

stan::agrad::var::vi\_

vari \* vi\_

Pointer to the implementation of this variable.

**Definition:** var.hpp:40

unlikely

#define unlikely(x)

**Definition:** likely.hpp:9

var.hpp

stan::agrad::var

Independent (input) and dependent (output) variables for gradients.

**Definition:** var.hpp:27

vv\_vari.hpp


---

[ Stan Home Page ]

*© 2011–2014,
Stan Development Team.*
